# Possible mechanism of action potential propagation mediated by static electric field: A novel assumption of understanding nerve interaction and ephaptic coupling

**DOI:** 10.1016/j.heliyon.2024.e37637

**Published:** 2024-09-10

**Authors:** Y.M. Guo, C.K. Ong

**Affiliations:** a Department of Physics, Xiamen University Malaysia, Jalan Sunsuria, Bandar Sunsuria, Sepang, 43900, Selangor, Malaysia; b Department of Physics, National University of Singapore, 2, Science Drive, 117551, Singapore; c The Key Laboratory for Magnetism and Magnetic Materials of the Ministry of Education, School of Physical Science and Technology, Lanzhou University, Lanzhou, 730000, China

## Abstract

The generation and propagation of physical signals in living biosystems are continuous issues. Traditional Hodgkin-Huxley model based on ionic current conduction could not explain the fast transmission of action potential in myelinated axons and factors influencing action potential velocity. We propose that the ion flow induced by $Na_{v}$ channel generates near field quasi-static electric field at extracellular space, termed as an ephaptic field which is able to excite nearby passive axons. Our simulation indicates that the static electric field produced by sodium ion channels in one node of Ranvier is improbable to stimulate the ion channels in the adjacent neighboring node. However, the ion channel ring in one node of Ranvier could induce the shift of membrane potential (0.01 mV) on the node at nearby axons (100 μm) in a bundle of axon synchronously, suggesting zig-zag propagation of action potential. Together with the superposition effect of ephaptic feedback field generated by the synchronized movement of adjacent parallel axons stimulate the adjacent node of the original axon, strengthen the action potential to travel in a zig-zag pattern. Our model also provides an explanation for the rapid velocity of action potential propagation reported in experimental studies.

## 1 Introduction

The generation and propagation of nerve signals, often known as action potential(AP) or voltage spikes, is a subject of continuing research in the field of neurological systems. The formation of AP occurs locally within ion channel pores, in close proximity to the boundary of chaos. The voltage spike occurs as a result of the shift in ion flow from states of high conductance to levels of low conductance. The Hodgkin-Huxley (HH) hypothesis, which relies on the flow of electrochemical currents, effectively elucidates and forecasts the observations in unmyelinated axons [1]. Nevertheless, the problem regarding the propagation of the signal along myelinated axons remained unresolved. The Hodgkin-Huxley theory, which is based on the local movement of ions along the axon, fails to account for the long-range transmission of APs across myelin sheaths and the rapid pace of propagation. The application of soliton theory to mechanical wave propagation, which relies on phase changes, faces several challenges. One such challenge is that the time it takes for the soliton to propagate does not align with the experimental data or the time required for ion channels to open. This discrepancy involves both the displacement of channel segments and the time it takes for ion circulation to initiate and generate a new AP [2], [3].

**Figure 1:**
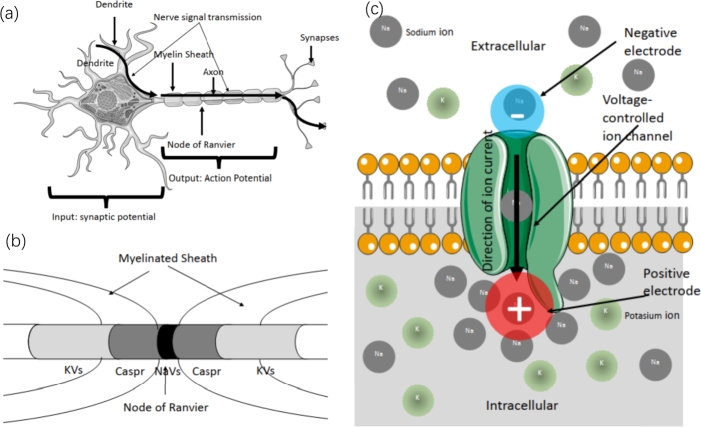
(a) The schematic graph of a motor neuron shows that it can be divided into dendrites, axons, and synapses. The nerve signal is transmitted from the dendrites through the axon and reaches the synapses. (b) Myelinated axons are mainly divided into three sections, including the nodes of Ranvier (Black), paranodes (Dark grey), juxtaparanodes (Light grey), and internodes. Each contains different specific domains. The Na_v_s mainly distributed in the node of Ranvier, K_v_s in juxtaparanodes and Caspr protein in paranodes. [13] (c) The schematic diagram reveals an activated sodium channel on the surface of the membrane; the upper side is extracellular fluid, and the lower side is intracellular fluid. The open sodium channel selectively allows sodium channel ions to flow into the intracellular fluid due to the chemical potential. The rapid ion inflow accumulates on the intracellular, generating an electric dipole with an intracellular side positive.

We first describe briefly the functional domains of motor neurons for instance. As sketched in Fig. 1(a). The dendrite terminals receive signals and generate postsynaptic potentials, which facilitate the influx of ions and depolarize the cell's rest potential. The amplitude of the postsynaptic potential is diminutive and falls beneath the threshold potential required for an AP. Nevertheless, every each neuron cell is composed of over 1000 terminals. The signal will be transmitted to the axon hillock, a region that connects the soma and axon. This region contains a high concentration of ion channels and is responsible for initiating the AP [4]. AP will occur in the axon and will be further elaborated upon at a later point.

In nature, certain axons are surrounded by myelinated sheaths, which contain periodic gaps known as nodes of Ranvier, as depicted in Fig. 1(a). The majority of voltage-gated ion channels and pumps are concentrated at these nodes [5]. Within the node of Ranvier of a myelinated axon, the voltage-controlled sodium ion channels ($Na_{v}$s in Fig. 1(a)) are located at the center, while the voltage-controlled potassium channels ($K_{v}$s in Fig. 1(a)) are positioned on either side in regions known as juxtaparanodes. The area between these channel zones, termed paranodes, predominantly contains Caspr, a protein family crucial for the development of myelinated axons. As shown in Fig. 1(b), these proteins form a ring around the circumference of axons. There are few ion channels within the myelinated sheath region, resulting in minimal ion flow through the membrane and no transient change in membrane potential to propagate APs. Consequently, APs propagate via “saltatory” conduction in the ion current perspective of the Hodgkin-Huxley (HH) model. However, the ion current theory does not account for the absence of AP in the myelinated sheath and the mechanism of saltatory conduction. Recent innovative proposals have sought to elucidate the mechanism of rapid signal transmission through the axon. These include: (1) modeling AP propagation based on electromagnetic (EM) waves through a waveguide [6], [2]; (2) intracellular electric fields [3]; (3) quantum confined ion superfluid [7]; (4) cell vibron polariton [8]; and (5) water molecules on the neuron surface [9].

Recent research has focused on ephaptic interactions in spike propagation among a group of axons. This research has discovered that the extracellular potential also significantly influences the propagation of AP. It has been observed that the extracellular potential alters the speed of conduction [10] and contributes in synchronized firing [11]. The impact of the extracellular electrical characteristics can also be examined using modified cable theory, which elucidates the increase in conduction velocity through ephaptic coupling and the enhancement of energy efficiency in propagation. Additionally, other factors can influence propagation [12].

We suggest a signaling transmission method facilitated by the ephaptic field through adjacent passive parallel axons in the extracellular region. The opening and shutting of voltage-gated ion channels create dipole oscillations that produce an ephaptic field. This field depolarizes passive axons in the vicinity through the extracellular region and triggers the activation of channels in those axons. The adjacent axons will replicate and produce the ephaptic field, so stimulating the subsequent node in the original axons through a feedback mechanism.

## 2 Result

The abbreviation of the parameters concerned is listed in Table 1.

**Table 1:** List of abbreviation.

Notation	Meaning
*r*	Radius of axon
*d*	Inter-nodal distance
*l*	Length of the node of Ranvier
$\rho_{Na_{v}}$	Number density of voltage-controlled Na_v_ channel
*I*_*Na*_	The ion current inflow cross membrane through a Na_v_ channel
*ϵ*_*r*_	Relative dielectric constant
**p**(**t**)	Dipole moment of single Na_v_ channel
Δ*q*	The accumulation charge in a dipole
*N*	Number of the ion channel in Sodium channel zone

### 2.1 Collective electric field from dipoles generated by ion channels

The influx of sodium ions from the extracellular fluid to the intracellular fluid through the opening $Na_{v}$ channels, driven by chemical potential, causes localized potential variation across the membrane. This variation arises from the redistribution of ions on a microscopic scale, a process known as depolarization in a macroscopic view, which can be measured experimentally [1]. Focusing on each individual $Na_{v}$ channel, as illustrated in Fig. 1(c), $Na^{+}$ ions flow from the extracellular region to the intracellular region due to the concentration gradient or chemical potential. The collective electric field generated by the dipoles, induced by the number of ion channels in the $Na_{v}$ region, creates a strong electric field. We detail the simulation model used to quantify the magnitude and potential of this electric field.

Assume that axons are arranged in parallel with each other in a portion of the myelinated nervous system. These neurons are considered to be regular cylinders with a radius of r=0.5 μm [14] and an inter-axon distance of approximately d=20nm [15].

**Figure 2:**
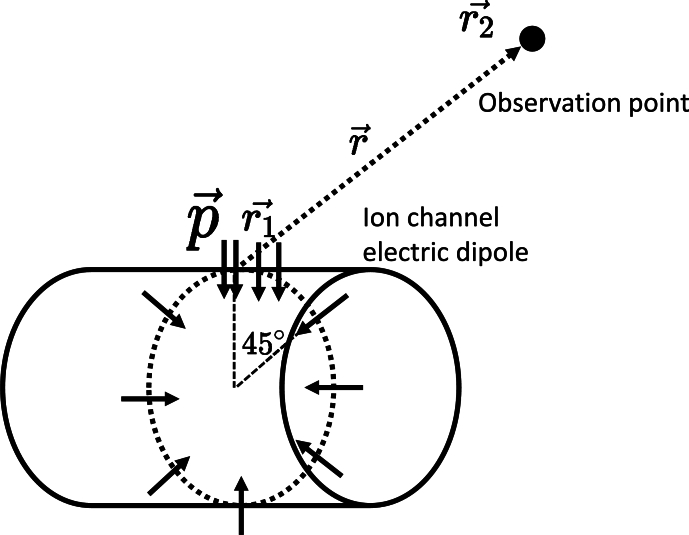
The schematic diagram of the approximated distribution of the ion channel (so as the electric dipole). Eight arrays of ion channels arranged evenly on the surface of the column, the inclined angle of 45^∘^, $\overset{\to }{r_{2}}$ represents the location of the observation point, $\overset{\to }{r_{1}}$ represents the source point of the electric dipole on the ion channel.

The $Na_{v}$ channels are mainly distributed at the center of the node of Ranvier. $K_{v}$ channels are distributed on both sides of the node of Ranvier, each node of Ranvier has a length of l=1 μm [14], with the density of $Na_{v}$ channels [16], *ρ* equal to $1000{\;\mu \text{m}}^{-2}$ [17]. Each $Na_{v}$ channel has peak current of $I_{\mathrm{Na}}=60\text{ pA}$ [18] with duration of 2 ms [19]. The transmembrane electric potential difference and chemical potential cross-membrane influence the flow of sodium ions simultaneously. The electric field drives sodium ions to flow out through the ion channel, and the diffusion chemical potential force inhibits the flow of sodium ions. The balance between membrane electric potential difference and chemical potential will arise after 2 ms, determined by the experiment [19]. The extracellular fluid has the relative dielectric constant of $\varepsilon_{r}=60$ [20]. In principle we need to sum over all ion channels encircling the node to calculate the total electric field as equation (1), by considering symmetry, with the aid of trial and error, we found that including 8 equal-spaced arrays are converging with 16 equal-spaced array calculation. This model assumes that the ion channel is evenly distributed throughout 8 arrays that are placed equally apart, as shown in Fig. 2. The aggregate electric field can be determined by summing the electric fields produced by the electric dipoles formed by the clusters of ion channels. This can be written as [21]:

(1) $$E(\overset{\to }{r})=\sum_{\mathrm{ionchannel}}\frac{3\cdot \overset{ˆ}{r}(p(t)\cdot \overset{ˆ}{r})-p(t)}{|\overset{\to }{r}{|}^{3}}$$

This formula is a modification of the formula for the electric field generated by an electric dipole, where $\overset{\to }{r}$ represent the vector pointing to the observation point from the position of the dipole, as in Fig. 2, $\overset{\to }{r}={\overset{\to }{r}}_{2}-{\overset{\to }{r}}_{1}$, where ${\overset{\to }{r}}_{1}$ represents the spatial location of the electric dipole, and ${\overset{\to }{r}}_{2}$ represents the spatial location of the observation points of electric field; represents the unit vector of $\overset{\to }{r},\overset{ˆ}{r}=\frac{\overset{\to }{r}}{\left|\overset{\to }{r}\right|};p(t)$ represents the ion channel dipole, which will be discussed in detail in following.

For each activated $Na_{v}$ channel, we assume that within 2 ms, a significant influx of sodium ions into the membrane occurs, resulting in the formation of a positive electrode composed of sodium ions and a negative electrode due to the absence of sodium ions. This process creates a cross-membrane electric dipole, as illustrated in Fig. 1 (c). The accumulated charge, considered as the dipole charge, can be calculated by the product of the ion current intensity and the accumulated time, $\Delta q=I_{\mathrm{ion}}\cdot \Delta t$, where Δ*q* is the accumulated charge during Δ*t*, the time the ion channel remains open. The distance between the electric dipole is approximately equal to the thickness of the cell membrane, $d_{\mathrm{membrane}}$. Therefore, the dipole moment of a single ion channel can be expressed as:

(2) $$p(t)=d_{\text{membrane }}\Delta q\overset{ˆ}{k},$$

where $\overset{ˆ}{k}$ represents the unit vector with the direction of the dipole, pointing inward from extracellular to intracellular, perpendicular to the surface of the membrane. In our work, we primarily consider using quasi-static model to calculate the electric field in the extracellular region assuming the slow time varying of ion channel polarization [21]. Consequently, the contribution of magnetic field is neglected, since dynamics contribution by magnetic field is relatively small.

Ghosh and its colleagues have suggested that voltage-controlled ion channels exhibit cooperatively through their interaction in generating APs [22], [23]. This interaction results in changes in the amplitude of APs, hyperpolarization, and a rapid onset of APs. Cooperativity of ion channels, which is not included explicitly in our model, accelerate the generation of action potential. Here, we assume that all $Na_{v}$ channels open simultaneously due to this cooperative effect. For computational convenience, we employ a near-field approximation, assuming each channel acts as a single electric dipole and combining all channels in an array into a single total electric dipole. We calculate the total number of ion channels *N* in the sodium zone as:

(3) $$N=2\rho_{\text{channel}}\pi rl,$$

which is equal to the number of the electric dipole on the node of Ranvier, $\rho_{\text{channel}}=1000{\;\mu \text{m}}^{-2}$ is the number density of $Na_{v}$ channels on the node of Ranvier, r=0.5 μm is the radius of the node of Ranvier, l=1 μm is the length of the node of Ranvier. Therefore on average, each array occupied the number of the ion channel as $N_{\text{line }}=N/8\approx 393$, and the merged electric dipole for each array could be computed as, ${\overset{\to }{p}}_{\text{line }}=\overset{\to }{p}N_{\text{line }}$, where ${\overset{\to }{p}}_{line}$ is the merged dipole moment for each line.

### 2.2 Electric field computation under the collective motion of ion channels

**Figure 3:**
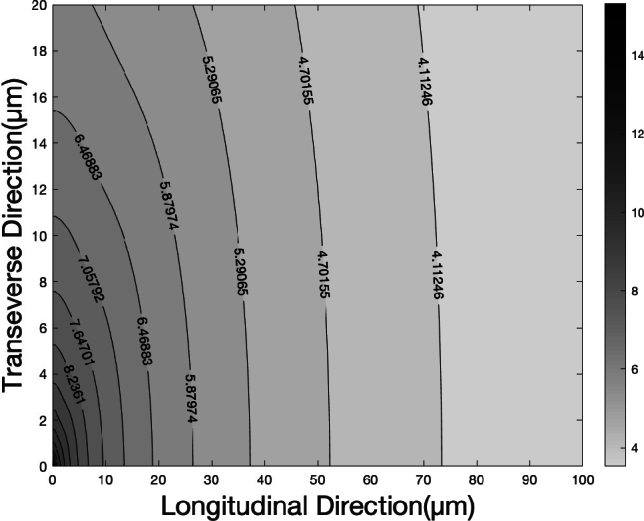
Distribution of the magnitude of the electric field with the center of ion channel ring located at the center of origin. The diagram indicates the electric field in the transverse direction starts from the surface of the membrane (0-20 μm) and longitudinal direction (0-100 μm). The magnitude of electric field is presented as logarithm of 10, in the unit of mV/m.

The simulation results, shown in Fig. 3, depict the electric field distribution in the extracellular region surrounded by axons. The results indicate that the electric field decays rapidly following a dipole pattern with distance, which aligns with the findings of Hale [24] and Chawla [25]. The field strength becomes negligible at the nearest node, approximately 100 μm away [14]. Additionally, the experimental values for internode length vary significantly; Arancibia-Carcamo's measurements range from 27 μm to 150 μm in the cortex, while another report indicates lengths exceeding 0.1 mm [26]. Our results clearly suggest that a direct approach is unlikely to activate the next node across the myelinated sheath. Therefore, we propose an indirect method for the electric field generated by ion channels to activate the next node for propagation.

### 2.3 Induced membrane potential difference sufficient to activate node on nearby axon

**Figure 4:**
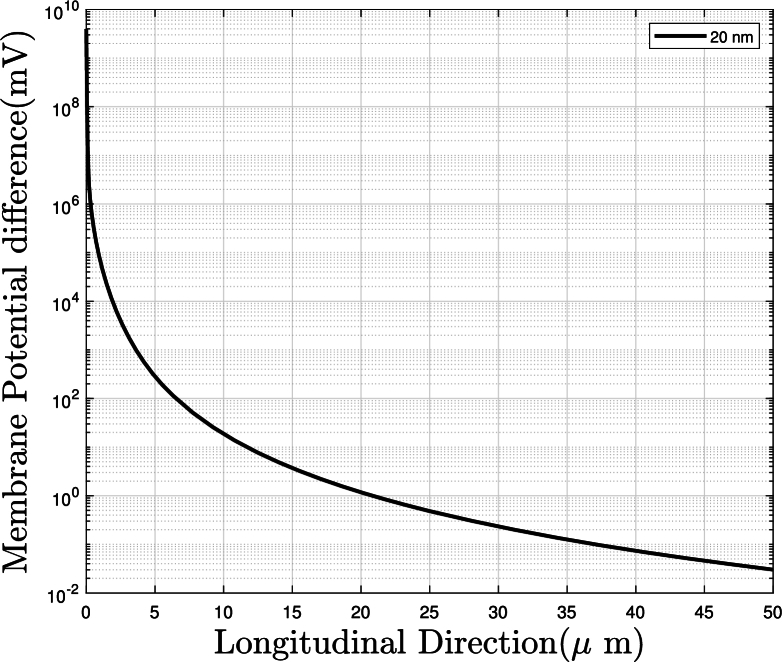
Variation of magnitude of membrane potential difference on a nearby passive parallel axon with distance along longitudinal (AP propagation) direction. The distance between two parallel axons in 20 nm. The quasi-static field is generated by Na_v_ channel of single node excited for 20 ms. The membrane potential difference decrease in AP propagation direction, and has a value of 0.01 mV at 50 μm.

**Figure 5:**
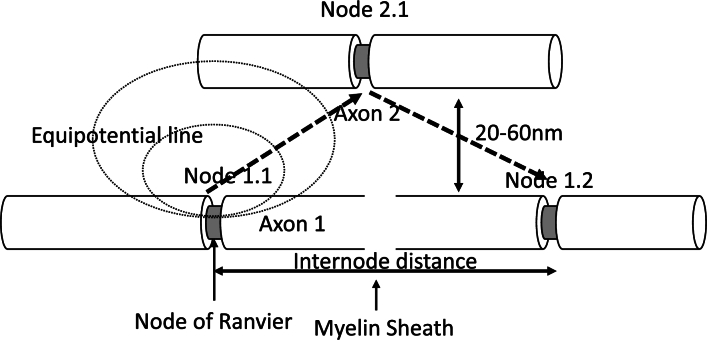
Two parallel aligned axons, axon 1 is active, and axon 2 is passive. Node1.1 at axon 1 is firing, generating an endogenous field at the extracellular region. This ephaptic field depolarizes the AP of axon 2 and the firing of node 2.1 when its value is near the sub-threshold. Subsequently, the AP of node 1,2 will be fired and propagated along a zigzag path between the neighbor axon. The propagation can be extended to more axons.

We next calculate induced membrane potential for channel located at a parallel axon by $U=-\int_{membrane}\overset{\to }{E}\cdot d\overset{\to }{l}$. In Fig. 3, we calculated the quasi-electric field generated by active channel at Node 1.1 as shown in Fig. 5 after 2 ms of excitation and hence membrane potential difference *U* induced on passive channel at Node 2.1, which has 20 nm far transverse distance of 50 μm to the AP propagatio direction from the active axon. The variation of *U* in propagation (longitudinal) direction is reported in Fig. 4: the curve decays fast initially and slow after 10 μm and reach a value of 0.01 mV at distance of 50 μm. Our calculation presented in Fig. 4 is contributed from one node and they are 40,000−50,000 neurons per ${\text{mm}}^{3}$ of a nervous system [24]. Considering superposition principle to the electric field generated by neurons will amplify the induced field and increase the membrane potential difference, providing sufficient voltage to activate ion channels for further propagation.

### 2.4 Ephaptic mediated propagation of AP

We propose that the activation of ion channels generates an ephaptic field, which plays an important role in the propagation of AP. The ephaptic field could be regarded as the quasi-static electric field, which could induce the potential difference across membrane on the neighbor passive axon when the strength of the field at the node of Ranvier of the neighbor passive axon is more significant than 0.01mV cross the $Na_{v}$ channels [27], which can activate sodium ion channels' threshold again and induce the depolarization process. The passive axon generates a secondary ephaptic field, which might potentially contribute to the existing ephaptic feedback field. This can lead to the depolarization of the Ranvier node in the original active axon and the activation of its sodium ion channels. Neighboring axons guide the transmission of the “saltatory” AP by generating and accepting ephaptic fields in a recurring cycle. This is illustrated in Fig. 5 for two axons, indicating a zig-zag pattern of propagation in analogy with conventional electromagnetic waveguides used in communication.

### 2.5 Propagation velocity and time by ephaptic field

**Figure 6:**
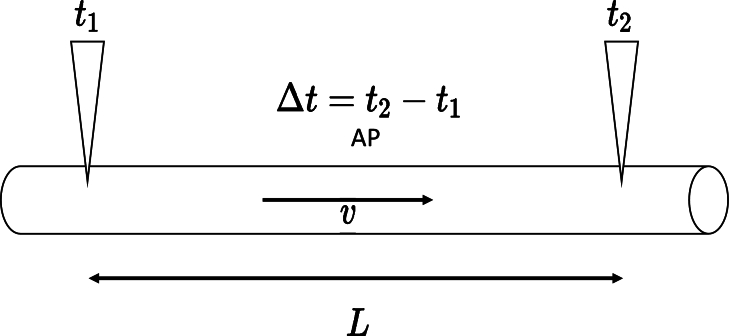
Schematic diagram indicates the measurement of propagation velocity of neuron, triangles represents the electrodes used for detecting the voltage spike and the time.

Next, we discuss the consistency of observed propagation velocity in neurons with our model. In the experiment, we determine the propagation of AP by measuring the time difference between the AP at two axon positions using electrodes. The propagation velocity *v* could be measured using v=L/Δt, as shown in Fig. 6., where *L* is the distance between electrodes and Δ*t* is the total time difference between electrode detected APs [28], [29]. We calculate the time consumption from one node to another in Ranvier. We assume that all the myelins have the same length and that there are N myelins between each electrode. The internodal distance is written as $L=NL_{in}$, where $L_{in}$ represents the length of the internode. Therefore, the time propagated per internode, *τ*, could be computed as τ=Δt/N, also named relay time in some papers [6]. The experimental result shows that the relay time is less than 100 micros. [6] In the conventional model, signal propagation is explained as the electric field driving the drift of the $Na^{+}$ in axons [30]. However, the ionic migration velocity within a solution is very slow, usually far less than 1.0mm/s. The relay time will be in the magnitude of 0.1 s, which is inconsistent with the result given in the experiment. In our model, the relay time primarily focuses on the accumulation of sufficient charge dipoles from ion channel rings, which generate an electric field that activates ion channels on parallel neighbors; the transmission time of this electric field is negligible. In the propagation process, we need at least two accumulation events for a signal to jump from one node to its nearest node.

From experiment [6], the time for AP to reach maximum from the threshold will take less than 500 μs to raise its voltage from threshold to peak by 40 mV. The field created by axons can be greatly increased by the superposition principle when fascicles, which are groups of axons, are taken into account. This is because axons are arranged parallel and densely, especially in the peripheral nervous system. These fascicles typically encompass around 20 axons [31], [32]. The request accumulation time for the dipole to generate potential near the threshold potential to excite the neighbor node could be much less than the peak value. For example, if we consider there are 10 axons in the firing channel, the accumulation time required for the AP to excite the next node would be 500 μs/10=50 μs, which is in the order of relay time reported [6].

## 3 Discussion

**Figure 7:**
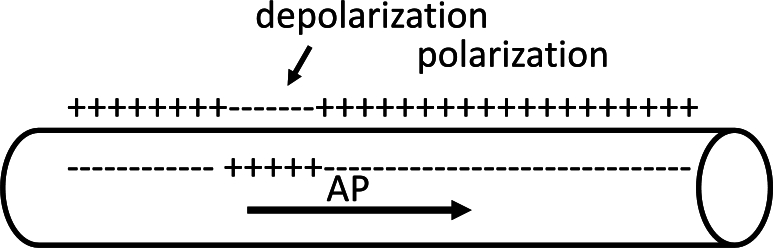
The propagation of nerve signal in unmyelinated axons. The signal propagated through the transmission of polarization and depolarization.

AP propagation through ephaptic field cannot be established in unmyelinated axon. The ion channel, both the $Na_{v}$ channel and the $K_{v}$ channel, is distributed uniformly on the surface of the unmyelinated axon instead of being distributed in rings as in myelinated neurons. The density of the ion channel [33] is $5-50{\;\mu \text{m}}^{-2}$, lower than that in myelinated axons. Fig. 7 explains the propagation of AP via polarization and depolarization based on the HH model in an unmyelinated axon [1]. Activation of a sodium ion channel induces positive polarization inside the membrane. The open and closing of $Na_{v}$ channels, as well as the open of $K_{v}$ channels, drive AP propagation by alternating polarization and depolarization along the axon. Therefore, the polarization and depolarization in the unmyelinated axon create a net electric field that cannot depolarize the neighboring axon. In short, the unmyelinated axon cannot establish any feedback from the ephaptic field.

In the case of myelinated axons, which has higher density voltage-controlled ion channels than unmyelinated axons, as their ion channels are clustered at the node of Ranvier. The electric field generated from channels in a node cannot activate channels in the next node on the same axon. However, the superposition of multiple ion channels enables the active axon's channels to generate an ephaptic field strong enough to activate channels on parallel axons.

In the generation and propagation of the AP spike, Nav, Kv and Cl channels play different roles. Kv channels play a predominant role in the repolarization process by restoring membrane potential, generating the counteracting electric field to restore equilibrium, and preparing for the next spike [34]. The stimulation of depolarization by Nav channels, restoration by Kv channels in the repolarization process and stabilization of resting potential by Cl channels contribute to the overall propagation of action potential.

## 4 Conclusion

In conclusion, we propose a novel theoretical model to explain the saltatory propagation of the AP in the myelinated axon mediated by an electric field. The endogenous field generated by induced dipoles on the ion channel is distributed in rings at the node of Ranvier. The strength of the endogenous field in the extracellular region at a distance of 50 μm is of the order of 0.01 mV and is recognized as an ephaptic field. We further suggest that Nav channels open as the membrane voltage reaches the threshold, causing depolarization on the surface of the nearby axon. The rapid flow of sodium ions then generates electric dipole field, which propagates to more nearby axons, thereby facilitating the propagation of APs. The ephaptic field feedback facilitates the propagation of the AP in axons. The proposed study sheds some light on the mechanism of AP propagation.

## CRediT authorship contribution statement

**Y.M. Guo:** Writing – review & editing, Writing – original draft, Visualization, Validation, Project administration, Investigation, Data curation, Conceptualization. **C.K. Ong:** Writing – review & editing, Writing – original draft, Supervision, Funding acquisition, Formal analysis.

## Declaration of Competing Interest

The authors declare the following financial interests/personal relationships which may be considered as potential competing interests: C.K. Ong reports financial support was provided by Xiamen University - Malaysia. If there are other authors, they declare that they have no known competing financial interests or personal relationships that could have appeared to influence the work reported in this paper.

## Data Availability

The data that support the findings of this study are available from the corresponding author, Prof. Ong, upon reasonable request.
